# Modulation of α-Enolase Post-Translational Modifications by Dengue Virus: Increased Secretion of the Basic Isoforms in Infected Hepatic Cells

**DOI:** 10.1371/journal.pone.0088314

**Published:** 2014-08-29

**Authors:** Luiza M. Higa, Bruno M. Curi, Renato S. Aguiar, Cynthia C. Cardoso, André G. De Lorenzi, Silvia L. F. Sena, Russolina B. Zingali, Andrea T. Da Poian

**Affiliations:** 1 Instituto de Bioquímica Médica Leopoldo de Meis, Universidade Federal do Rio de Janeiro, Rio de Janeiro, Rio de Janeiro, Brazil; 2 Departamento de Genética, Instituto de Biologia, Universidade Federal do Rio de Janeiro, Rio de Janeiro, Rio de Janeiro, Brazil; 3 Clínica de Doenças Infecciosas e Parasitárias, Hospital Naval Marcílio Dias, Rio de Janeiro, Rio de Janeiro, Brazil; University of California Merced, United States of America

## Abstract

Hepatic cells are major sites of dengue virus (DENV) replication and liver injury constitutes a characteristic of severe forms of dengue. The role of hepatic cells in dengue pathogenesis is not well established, but since hepatocytes are the major source of plasma proteins, changes in protein secretion by these cells during infection might contribute to disease progression. Previously, we showed that DENV infection alters the secretion pattern of hepatic HepG2 cells, with α-enolase appearing as one of the major proteins secreted in higher levels by infected cells. ELISA analysis demonstrated that DENV infection modulates α-enolase secretion in HepG2 cells in a dose-dependent manner, but has no effect on its gene expression and on the intracellular content of the protein as assessed by PCR and western blot analyses, respectively. Two-dimensional western blots showed that both intracellular and secreted forms of α-enolase appear as five spots, revealing α-enolase isoforms with similar molecular weights but distinct isoeletric points. Remarkably, quantification of each spot content revealed that DENV infection shifts the isoform distribution pattern of secreted α-enolase towards the basic isoforms, whereas the intracellular protein remains unaltered, suggesting that post-translational modifications might be involved in α-enolase secretion by infected cells. These findings provide new insights into the mechanisms underlying α-enolase secretion by hepatic cells and its relationship with the role of liver in dengue pathogenesis. In addition, preliminary results obtained with plasma samples from DENV-infected patients suggest an association between plasma levels of α-enolase and disease severity. Since α-enolase binds plasminogen and modulates its activation, it is plausible to speculate the association of the increase in α-enolase secretion by infected hepatic cells with the haemostatic dysfunction observed in dengue patients including the promotion of fibrinolysis and vascular permeability alterations.

## Introduction

Dengue is the most prevalent arthropod-borne viral disease, with 2.5 billion people living in risk areas. This disease is caused by dengue virus (DENV), a member of the *Flaviviridae* family, which also comprises several medically-important viruses, such as West Nile virus (WNV), yellow fever virus (YFV) and hepatitis C virus (HCV). A recent study estimated that DENV infects 390 million individuals annually, resulting in 22,000 deaths in tropical and subtropical areas worldwide [1]–[2], but despite this global public health burden of dengue, there is neither prophylactic nor therapeutic vaccine available and therapy consists only in supportive treatment.

DENV infection is often asymptomatic, but it can also lead to clinical manifestations ranging from a mild febrile illness to a severe and potential life-threatening disease. To differentiate the degree of disease severity, World Health Organization (WHO) classifies dengue into dengue fever (DF), dengue hemorrhagic fever (DHF) and dengue shock syndrome (DSS) [3]. Recently, a new classification has been proposed to facilitate clinical management, categorizing the disease into dengue without warning signs, dengue with warning signs and severe dengue [4]. Criteria for dengue with warning signs diagnosis include clinical fluid accumulation, mucosal bleed, liver enlargement and increased hemoconcentration concurrent with thrombocytopenia, while severe dengue is characterized by severe plasma leakage, hemorrhage and organ impairment. Liver enlargement and increase in the levels of plasma transaminases levels are also among the criteria to diagnose dengue with warning signs and severe dengue [4].

Hemostasis abnormalities, namely thrombocytopenia, increased vascular permeability, coagulopathy and abnormal fibrinolysis, have been frequently observed in dengue patients [5]–[8]. DHF patients present decreased plasma levels of fibrinogen and plasminogen, reduced α_2_-antiplasmin activity and an increase of fibrin degradation products, plasmin-antiplasmin complexes and tissue-type plasminogen activator (tPA), indicating that patients with life-threatening forms of dengue develop hyperfibrinolysis [5]–[14]. Excessive activation of fibrinolysis increases the tendency for hemorrhage, which is in agreement with the report that DENV-infected rhesus macaques presented hemorrhagic manifestations and elevated fibrinolysis products in plasma [15].

Recently, molecules secreted by DENV-infected cells have been associated with the pathogenesis of clinical manifestations [16]–[17], being an interesting and unexplored field for dengue research. In a previous study of the global effects of DENV infection on protein secretion by a hepatic cell line, our group identified α-enolase among the differentially-secreted proteins [18]. Enolase is found in all living organisms and is highly conserved across species [19]. In humans, three isozymes of enolase exist: α-enolase, β-enolase and γ-enolase, which are encoded by three different genes: ENO1, ENO2 and ENO3. α-enolase is expressed in almost all tissues, while β-enolase is found preferentially in muscle and γ-enolase is expressed in neurons and neuroendocrine tissues. Aside from its canonical enzymatic role in glycolysis and gluconeogenesis pathways, in which it catalyzes the dehydration of 2-phospho-D-glycerate (PGA) to phosphoenolpiruvate (PEP) and the reverse reaction of hydration of PEP to PGA, respectively, several other functions have been attributed to α-enolase [19]–[20]. ENO1 mRNA alternative stop codon produces a 37 kDa nuclear protein that binds c-myc P2 promoter and functions as a transcriptional suppressor [21]. α-enolase has also been associated with thermal tolerance and has been described as a heat shock protein (HSP48) in the *yeast Saccharomyces cerevisiae* [22] and a hypoxic stress protein in endothelial cells [23]. Moreover, α-enolase binds to plasminogen and regulates its activation [24]–[25]. Plasminogen activation mediated by α-enolase plays important roles in several physiological and pathophysiological processes, including tissue remodeling, inflammatory response, pathogen invasion and metastasis of tumor cells [19]–[20], [26]. Since dengue is an inflammatory disease characterized by haemostatic dysfunction and alterations in vascular permeability, we hypothesize that an increase in α-enolase secretion may contribute to the excessive fibrinolysis observed in dengue. In this work, we investigated the effects of DENV infection on α-enolase expression, secretion and post-translational modifications in HepG2 cells, a hepatic cell line. The potential physiological relevance of our results is supported by data obtained with plasma samples from dengue patients.

## Methods

### Cell lines and viruses

HepG2 cells were maintained in Dulbecco's modified Eagle medium (DMEM) containing 10% fetal bovine serum (FBS). BHK cells were cultured in α-MEM supplemented with 10% FBS. HepG2 and BHK cells were cultured at 37°C in a 5% CO_2_ humidified incubator. *Aedes albopictus* C6/36 cells (ATCC) were maintained at 28°C in Leibovitz L15 medium supplemented with 1.44 mM L-glutamine, 0.75 g/L sodium bicarbonate, 0.3% tryptose phosphate broth, non-essential amino acids and 5% fetal bovine serum.

For preparation of virus stock samples, DENV serotype 2 strain 16681 was propagated in C6/36 cells. Briefly, C6/36 cells were infected at multiplicity of infection (MOI) of 0.1. After 9 days, the conditioned medium containing the virus particles was harvested and centrifuged at 300× *g* for 10 min; 2,000× g for 10 min and 10,000× g for 30 min for live, dead cells and cellular debris removal respectively. Supernatants were collected and stored at −80°C. Virus titers were determined by plaque assay performed on BHK cells as described elsewhere [27].

### Plasma samples

Plasma samples were obtained from healthy individuals and dengue patients from Hospital Naval Marcílio Dias. Dengue cases were laboratory-confirmed by IgM and IgG-seroconversion (Pambio) and detection of NS1 antigen (Bio-Rad) analyzed by ELISA and/or detection of DENV RNA by RT-PCR. Dengue cases were classified in a binary fashion as being “DSS” or “not-DSS” following WHO recommendations [3]. Patients defined as DSS cases presented shock and severe organ involvement (liver, CNS or cardiovascular decompensation secondary to plasma leakage).

### Ethical statement

The experimental procedures with human plasma samples were approved by the ethical committee of the Hospital Naval Marcílio Dias (13579013.5.0000.5256), Rio de Janeiro. Written informed consent was obtained from all the patients upon enrollment.

### Cell infection and sample processing

HepG2 cells were seeded and maintained for 48 hours in DMEM containing 10% FBS. For infection, the growth medium was replaced by serum-free medium containing the virus at the indicated MOIs. Mock-infected cells were incubated with the conditioned media from uninfected C6/36 cells prepared exactly as performed for viral propagation. After 1 hour, cells were washed in PBS and incubated with serum-free DMEM for 24 hours. After this period, the conditioned media containing the secreted proteins were harvested and protease inhibitors (1 mM PMSF, 1 µM pepstatin and 10 µM leupeptin) were immediately added to avoid protein degradation. Conditioned medium samples were filtered (0.22 µm membrane) to remove cells and cellular debris and stored at −20°C for posterior analyses by ELISA, electrophoresis and western blotting. To perform 2D-eletrophoresis experiments, conditioned medium samples were concentrated as described elsewhere [18]. Briefly, conditioned medium concentration was carried out by ultrafiltration through a 10 kDa-cut off membrane in an Amicon system. Concentrated samples were lyophilized and solubilized in 250 µl deionized water. To remove contaminants, samples were treated with 2-D Clean-up Kit (GE Healthcare) according to manufacturer's instructions. The resulting pellet was dissolved in rehydration solution (7 M urea, 2 M thiourea, 100 mM DTT, 0.5% carrier ampholytes, 2% CHAPS, 0.002% bromophenol blue). Protein concentration was determined using 2-D Quant kit (GE Healthcare) as advised by the manufacturer.

To prepare cellular lysates, after conditioned medium collection, adherent cells were washed, harvested in ice-cold PBS and centrifuged at 400× g for 10 min at 4°C. Cells were disrupted by resuspending the cellular pellet in lysis buffer (7 M urea, 2 M thiourea and 2% CHAPS) containing 1 mM PMSF, 10 µM leupeptin, 1 µM pepstatin A, 100 µg/ml DNAse and 25 µg/ml RNAse. Samples were vortexed for 2 min and incubated on ice for 30 min. Cell lysates were sonicated on ice for 1 min with 5 s pulses (5 s pauses between pulses). Then, the samples were centrifuged at 16,000× g for 20 min at 4°C and the cleared supernatants were collected. Cell lysates were then treated with 2-D Clean-up Kit and protein concentration was determined using 2-D Quant kit (GE Healthcare) according to manufacturer's instructions.

To perform gel electrophoresis of plasma samples, albumin was removed through affinity chromatography using Cibacron Blue 3GA agarose resin (Sigma). Plasma samples (60 µl) were applied into 200 µl of the resin previously equilibrated with 0.01 M Tris, pH 7.5. After washing the unbound fraction, protein elution was performed by 1.5 M NaCl in 0.01 M Tris, pH 7.5. The resin was washed in equilibration buffer and the procedure was repeated to ascertain the removal of albumin. Protein concentration was determined by Bradford assay. To perform two-dimensional electrophoresis, albumin-depleted samples were treated with 2-D Clean-up Kit (GE Healthcare) and protein concentration was determined using 2-D Quant kit (GE Healthcare), according to manufacturer's instructions.

### Flow cytometry

Flow cytometry analysis was performed to assess the frequency of DENV-infected cells by detecting intracellular DENV antigens. Briefly, HepG2 cells were either mock-infected or infected with MOI = 1, 2 or 4. At 24 hours post-infection, HepG2 cells were harvested and fixed in 4% paraformaldehyde in PBS for 15 min. Cells were permeabilized with 0.1% saponin in PBS and nonspecific binding sites were blocked by incubating the cells with 3% bovine serum albumin (BSA) in PBS for 30 min at room temperature. Then, cells were incubated for one hour with DENV-reactive monoclonal antibody MAB8705 (Millipore) or an isotype control antibody, washed in PBS and labeled with an anti-mouse secondary antibody conjugated to Alexa fluor-488 (Invitrogen) for 30 min, followed by a PBS wash. Cells were subjected to flow cytometry analysis by using FACScalibur cytometer (Becton Dickson Immunocytometry System). For each sample, 10,000 events were acquired and analyzed using the CellQuest software. The background fluorescence was set using cells labeled only with the secondary antibody.

### ELISA

Conditioned medium and cell lysate samples (100 ng protein) from HepG2 cells mock-infected or DENV-infected were diluted in carbonate buffer (150 mM sodium carbonate and 350 mM sodium bicarbonate, pH 9.6) and adsorbed into 96 wells polystyrene plates (Corning) overnight at 4°C. Wells were washed in 0.05% Tween-20 in PBS (PBS-T) and nonspecific binding sites were blocked with 3% BSA in PBS-T for 2 h. Wells were washed with PBS-T and incubated with anti-α-enolase antibody (Santa Cruz Biotechnology) diluted in incubation buffer (1% BSA in PBS-T) for 1 hour at 37°C. Wells were then washed with PBS-T and incubated with horseradish peroxidase-conjugated secondary antibody for 1 hour at 37°C. Finally, wells were washed with PBS-T and incubated with chromogenic substrate OPD diluted in phosphate-citrate buffer with 0.012% hydrogen peroxide. To stop the reaction, 50 µl of a 3N HCl solution were added to each well. The optical densities were read in 490 nm using a microplate spectrophotometer Victor X (Perkin-Elmer). Serial dilutions of purified recombinant human α-enolase (Abcam) were used as standard for α-enolase quantification.

### Cell viability assays

Trypan blue exclusion and MTT (3-(4,5-dimethylthiazol-2-yl)-2,5-diphenyl tetrazolium bromide) reduction assays were used for determination of cell viability. Cells were plated at a density of 7.5×10^4^ cells per well in a 24-well plate and cultured exactly as for sample processing. For estimation of the percentage of viable cells by standard trypan blue dye exclusion assay, cells were washed with PBS and incubated with 0.25% trypsin, 1 mM EDTA in PBS to detach adherent cells, and 10 µl of the cellular suspension were added to 10 µl of 0.4% Trypan blue stain solution. Following a two min incubation, unstained live cells were counted on a hemocytometer. For MTT reduction assay, cells were washed in balanced salt solution, BSS (0.16 g/L Na_2_HPO_4_, 8 g/L NaCl, 0.4 g/L KCl, 0.016 g/L CaCl_2_, 0.154 g/L MgSO_4_ and 1.1 g/L glucose, pH 7,4) and 500 µl of 0.5 mg/ml MTT (USB Corporation) in BSS solution were added to each well. After two hours of incubation at 37°C, the supernatant was discarded and formazan crystals were dissolved in 500 µl of 0.04 M HCl in isopropanol solution. Absorbance was read at 570 nm and 650 nm for background correction.

### LDH release assay

To evaluate cell lysis, lactate dehydrogenase (LDH) release assay was carried out by using a Cytotox 96 NonRadioactive Cytotoxicity Assay kit (Promega) as advised by the manufacturer. Briefly, 50 µl of conditioned media from HepG2 cells were incubated with 50 µl of LDH substrate for 30 min and the reaction was stopped with 50 µl of 1N acetic acid. The optical densities were read in 490 nm using a microplate spectrophotometer Victor X (Perkin-Elmer). The percentage of cell lysis was calculated using a maximum LDH activity obtained for cells treated with 0.2% Triton X-100. Cellular lysis was quantified according to manufacturer's instructions.

### Real-time PCR

Total RNA from mock-infected or DENV- infected HepG2 cells was extracted using Trizol reagent (Life Technologies) according to the manufacturer's instructions. To prevent genomic DNA contamination, the samples were treated with DNAse I. Reverse transcription was performed using 2 µg of the RNA extracted from mock-treated or DENV-infected cells using High-Capacity cDNA Archive Kit (LifeTechnologies) and resulted in the synthesis of first strand cDNA. To analyze ENO1 gene expression, cDNAs were 5-fold diluted and 1 µl of each sample was submitted to real-time PCR using Power SYBR Green PCR Master mix (LifeTechnologies). The reaction was carried out using the following gene specific primers, sense 5′ ATG ACC CCA GCA GGT ACA TC 3′ and antisense 5′ CTG TGA GAT CAT CCC CCA CT 3′, and a StepOne Plus Real-time PCR system (LifeTechnologies). The samples were subjected to a first step at 95°C for 10 min followed by 40 amplification cycles consisting of DNA denaturation for 30 s at 95°C, and annealing of primers for 30 s at 60°C. The threshold cycles (Ct) for ENO1 and for the housekeeping gene (glyceraldehyde-3-phosphate dehydrogenase, GAPDH) were determined. The relative expression was calculated as described elsewhere [27].

### SDS-PAGE

Proteins from lysates of mock and DENV-infected cells (10 µg) and from albumin-depleted plasma samples (40 µg) from healthy donors (control), patients with non-severe dengue (not-DSS) and patients with dengue shock syndrome (DSS) were loaded into a denaturating polyacrilamide gel (SDS-PAGE) 10% in Tris-glicine buffer. Electrophoresis was carried out at 15 mA/gel using the electrophoresis Mini-Protean 3 system (Bio-Rad).

### Two-dimensional gel electrophoresis

Conditioned medium (100 µg), lysate (80 µg) or albumin-depleted plasma (80 µg) samples were diluted in rehydration solution (7 M urea, 2 M thiourea, 2% CHAPS, 100 mM DTT, 0.5% carrier ampholytes and 0.002% bromophenol blue). Samples were centrifuged for 10 min at 10,000× g and supernatants were collected and loaded into 7 cm-IPG strips with linear pH gradient from 3 to 10. Strips were covered with mineral oil to prevent urea crystallization and IPG gel strips dehydration. Isoeletric focusing (IEF) was performed at 20°C on an Ettan IPGphor platform (GE Healthcare). Initially, IPG strips were actively rehydrated for 12 h and then submitted to the following voltage profiles: 100 V for 1 h, 200 V for 1 h, 500 V for 1 h and finally, 3500 V for 4 h. Following IEF, the strips were incubated in equilibration buffer (75 mM Tris-HCl, 6 M urea, 29.3% glycerol, 2% SDS and 0.02% bromophenol blue) containing 1% DTT for 15 min and then another 15 min in 2.5% iodoacetamide in equilibration solution. For second dimension, focused IPG strips were placed on the top of a 12.5% polyacrylamide gel and sealed with agarose solution (0.5% agarose, 0.002% bromophenol blue in Tris-glycine buffer). SDS-PAGE was performed with 15 mA per gel for approximately 2 h. For visualization of proteins spots, gels were either stained with Coomassie blue or transferred to PVDF membranes to perform western blot analyses. For molecular weight determination, *PageRuler Prestained Protein Ladder* (Fermentas, Canada) was loaded into the gels. For gel staining, the gels were incubated in 2% phosphoric acid, 30% ethanol fixing solution for 2 h and then washed in 2% phosphoric acid. Gels were incubated with a solution containing 2% phosphoric acid, 18% methanol, 15% ammonium sulfate and 0.02% Coomassie blue G-250 for 72 h. To remove excessive staining, gels were washed with deionized water. Gels were stored in 1% acetic acid.

### Western blot

For Western blot analysis, proteins separated on either one- or two-dimensional polyacrylamide gels were transferred to PVDF membranes (Millipore) in a Trans-Blot^®^ SD Semi-Dry Transfer Cell system (Bio-Rad). Membranes were washed in 0.1% Tween in TBS (50 mM Tris, 150 mM NaCl), TBS-T. Nonspecific binding sites were blocked with 5% dry milk in TBS-T for 1 h. After blocking, the membranes were incubated with anti-α-enolase antibody for 1 h, washed in TBS-T and incubated with peroxidase-conjugated secondary antibody. Membranes were incubated with chemiluminescent substrate, ECL and exposed to x-ray hyperfilm for 1 min. For cell lysate samples, membranes were also probed with an anti-GAPDH antibody (Millipore) which was used to normalize protein loading. Blot images were captured using ImageScanner (GE Healthcare) and densitometry of protein bands or spots was performed and processed with Image J (NIH) and ImageMaster 2D Platinum software (GE Healthcare), respectively.

### Statistical analysis

Statistical analyses were performed using Graphpad Prism software (GraphPad software). All data were expressed as mean ± standard error of the mean (SEM). Statistical comparisons between mock and infected cells samples were performed by student's *t* test whereas comparisons among groups (mock-infected, MOI = 1, MOI = 2 and MOI = 4) were analyzed by repeated measures analysis of variance (ANOVA). Relationship between % DENV-infected cells and α-enolase content in conditioned medium was assessed by Spearman correlation. Data for α-enolase spots were analyzed by repeated measures two-way ANOVA with DENV infection and spots as factors. When ANOVA revealed a significant effect, the data were further analyzed with the Bonferroni or Dunnet *post hoc* test to determine specific group differences and to correct for multiple comparisons. Differences were considered statistically significant when *p*<0.05. Sample size was provided in the respective figure legends.

## Results

### DENV infection induces α-enolase secretion in HepG2 cells

**Figure 1:**
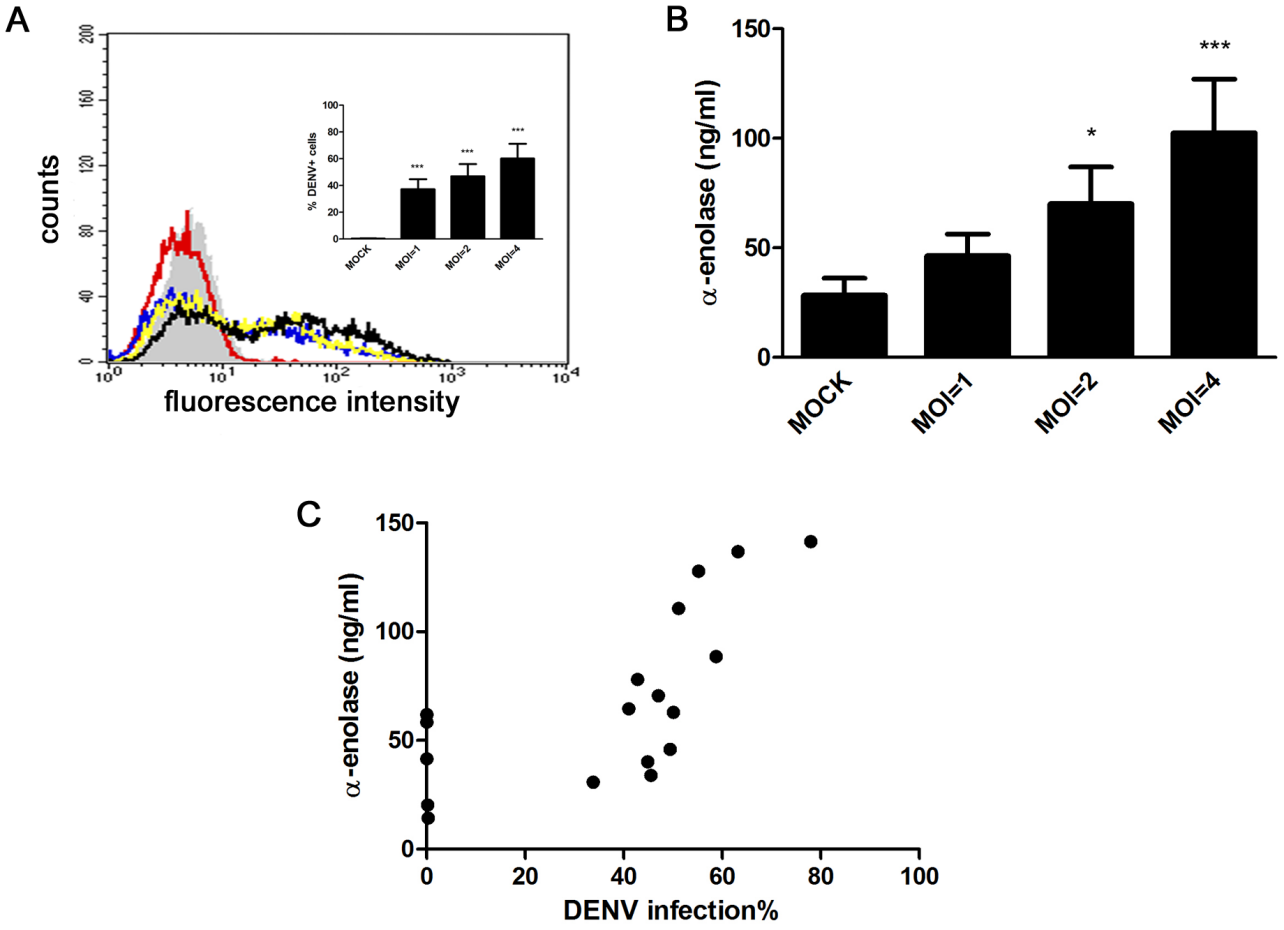
DENV infection induces α-enolase secretion in HepG2 cells. HepG2 cells were either mock-infected or infected with DENV (MOI = 1, 2 and 4) for 24 h. (A) DENV+ cells were measured by flow cytometry using an anti-DENV monoclonal antibody. Representative flow cytometry analysis of mock and DENV-infected HepG2 cells. Shaded histogram indicates DENV-infected HepG2 cells (MOI = 4) stained with an isotype control antibody, open histograms in red, blue, yellow and black show mock, DENV-infected cells with MOI = 1, MOI = 2 and MOI = 4, respectively. The inset graph shows the frequency of DENV+ cells and data represents mean ± SEM from 8 independent experiments. (B) α-enolase content in the conditioned medium was measured by ELISA. Graph shows mean ± SEM from 6 independent experiments. Differences between mock and DENV-infected cells were assessed using repeated measures ANOVA and Dunnet post-hoc test. Statistical significance compared with the control is indicated by asterisks (* *p*<0.05, *** *p*<0.001). (C) Correlation analysis between the frequency of DENV-infected cells and α-enolase content in the conditioned medium. The % infected cells was calculated by subtracting the mock-infected staining value from those obtained for infected cells. Relationship was assessed using Spearman correlation.

In a previous exploratory study on the secretome of DENV-infected HepG2 cells, we found α-enolase among the differentially-secreted proteins [18]. To further characterize the relationship between DENV infection and α-enolase secretion, we first sought to find a range of viral input that causes increasing viral infection in HepG2 cells. For this, we incubated the cells with DENV at multiplicities of infection (MOI) of 1, 2 and 4 for 24 h, stained for the presence of intracellular viral antigens, and subjected the cells to flow cytometry analysis. At these conditions, the percentage of viral antigens-positive cells consistently increased when cells were incubated with higher viral loads (Fig. 1A). Thus, using this range of MOI, we analyzed content of secreted α-enolase in the conditioned media of infected HepG2 cells. We observed that infected cells presented increased α-enolase levels when compared to mock-infected cells and that increasing MOI result in higher levels of secreted α-enolase (Fig. 1B). This result indicates that the infection modulates α-enolase secretion in a dose-dependent manner. Correlation analysis between the frequency of DENV-infected cells and α-enolase content in the conditioned medium revealed a significant positive correlation with a Spearman correlation coefficient r = 0.7046; p = 0.0005 (Fig. 1C).

**Figure 2:**
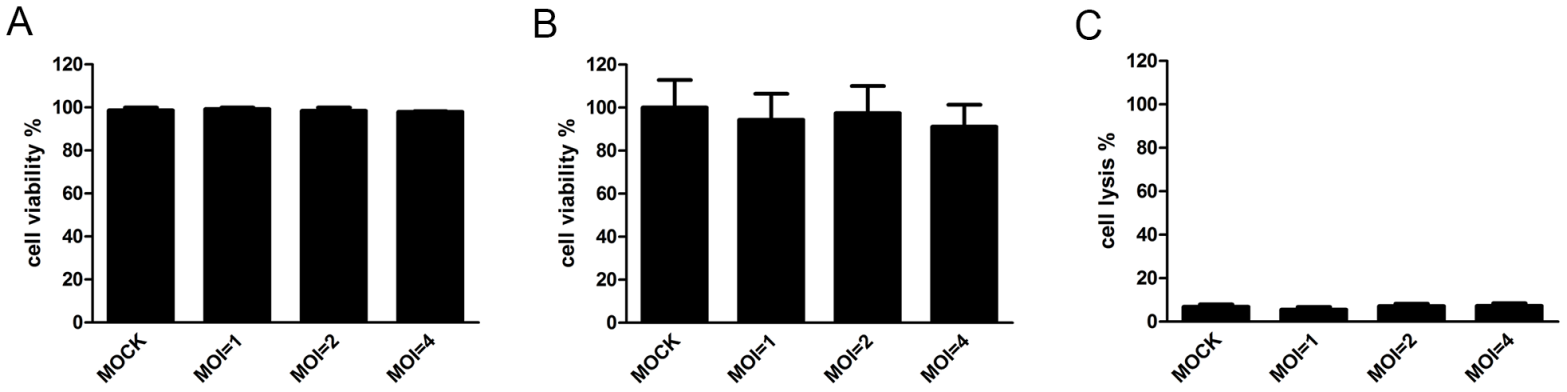
α-enolase in the conditioned medium of DENV-infected cells is not due to cell death. HepG2 cells were either mock-infected or infected with DENV (MOI = 1, 2 and 4) for 24 h. (A) Cell viability was measured by trypan blue exclusion assay. Graph shows mean ± SEM from 5 independent experiments. (B) Cell viability was assessed by MTT reduction assay. Graph shows mean ± SEM from 6 independent experiments. (C) Cell lysis was assessed by LDH release assay. Graph shows mean ± SEM from 5 independent experiments. Differences between samples from mock-infected and DENV-infected cells were assessed using repeated measures ANOVA test.

To certify that α-enolase observed in the conditioned medium was in fact secreted and not derived from cytoplasmatic contamination, we conducted cell viability assays. No significant differences were observed when mock-infected cells were compared to infected cells in Trypan blue exclusion (Fig. 2A), MTT reduction (Fig. 2B) and LDH activity assays (Fig. 2C). These data show that viability is not affected when cells are infected with MOI = 1, 2 or 4 for 24 h, suggesting that contamination of conditioned medium with cytoplasmatic proteins is not significant when compared to control cells.

### DENV infection induces a selective secretion of the more basic isoforms of α-enolase

**Figure 3:**
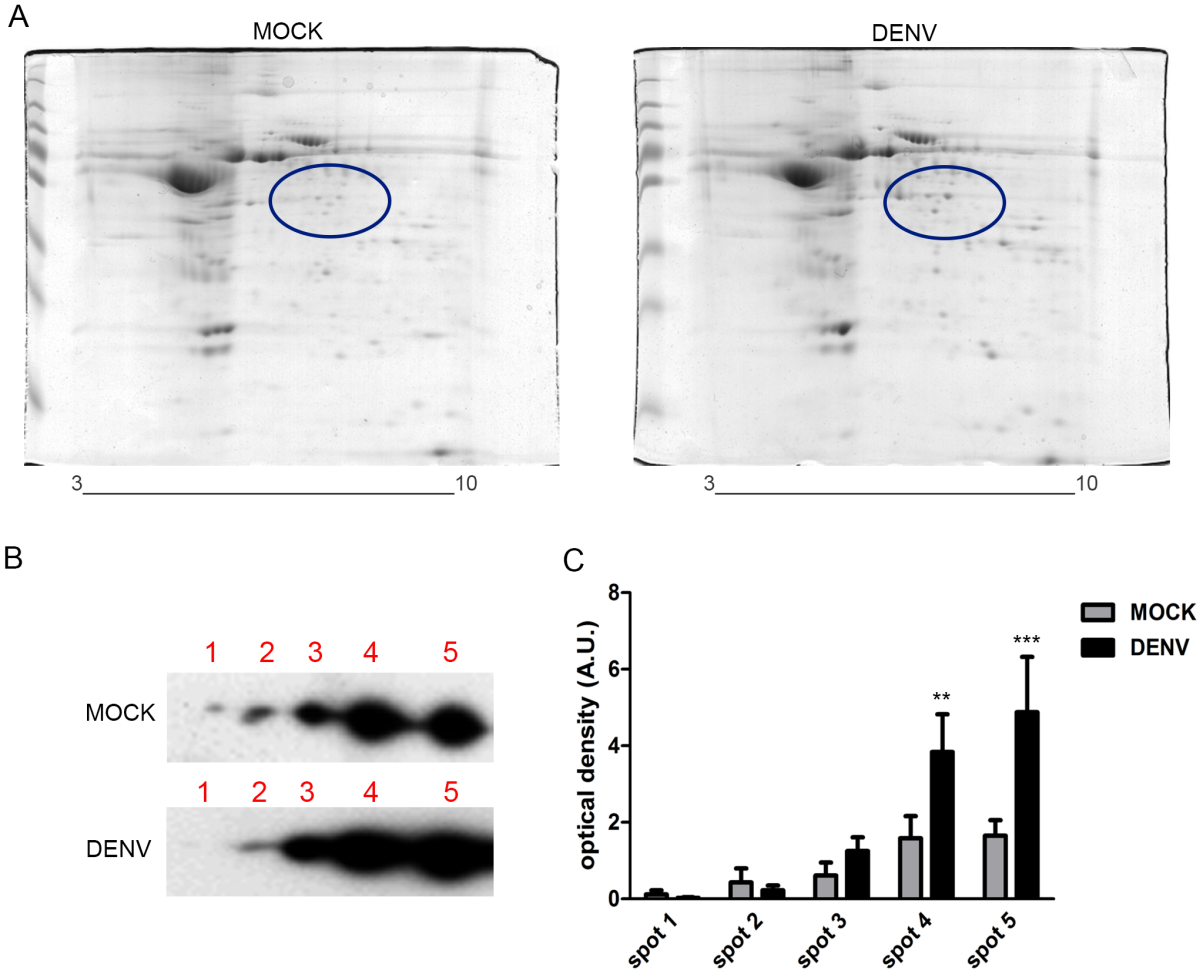
DENV infection induces changes in the distribution of secreted α-enolase isoforms. HepG2 cells were either mock-infected or infected with DENV (MOI = 4) for 24 h. Conditioned medium was collected and concentrated and the proteins were analyzed by two-dimensional gel electrophoresis (2-DE). (A) Gels stained with colloidal Coomassie blue. The circles indicate the region for α-enolase separation, which was selected accordingly to the theoretical molecular mass and isoeletric point of the protein. (B) Immunodetection of α-enolase isoforms on the 2-DE Western blot. α-enolase spots were detected in the region correspondent to the circles in Figure 3A. The figure shows a representative blot of 4 independent experiments (C) Densitometric analysis of α-enolase spots. Graph shows mean ± SEM from 4 independent experiments. Differences between spots from mock and DENV-infected cells were assessed using repeated measures two-way ANOVA followed by Bonferroni *post hoc* test. Statistical significance compared with the control is indicated by asterisks (***p<0.001,*p<0.001).

A number of post-translational modifications are shown to occur in α-enolase, including Ser phosphorylation, Lys acetylation and methylation of Asp and Glu residues, which results in distinct isoforms showing different isoeletric points [28]. To investigate whether these alterations were involved in the DENV infection-induced secretion, we performed two-dimensional electrophoresis (Fig. 3A) followed by Western blot for α-enolase detection (Fig. 3B). Western blot analyses revealed five spots with similar molecular weights but distinct isoeletric points, suggesting that at least five isoforms of α-enolase are secreted by mock and DENV-infected HepG2 cells. Densitometric analysis of the corresponding spots indicated a significant increase in the amount of the more basic isoforms (spots 4 and 5) secreted by DENV-infected cells. The more acidic isoforms, spots 1 and 2, appear to be slightly, though not significantly, decreased in samples from DENV-infected cells. These data reveal that DENV infection alters the distribution of α-enolase isoforms secreted by HepG2 cells.

### DENV infection does not alter the intracellular content of α-enolase

**Figure 4:**
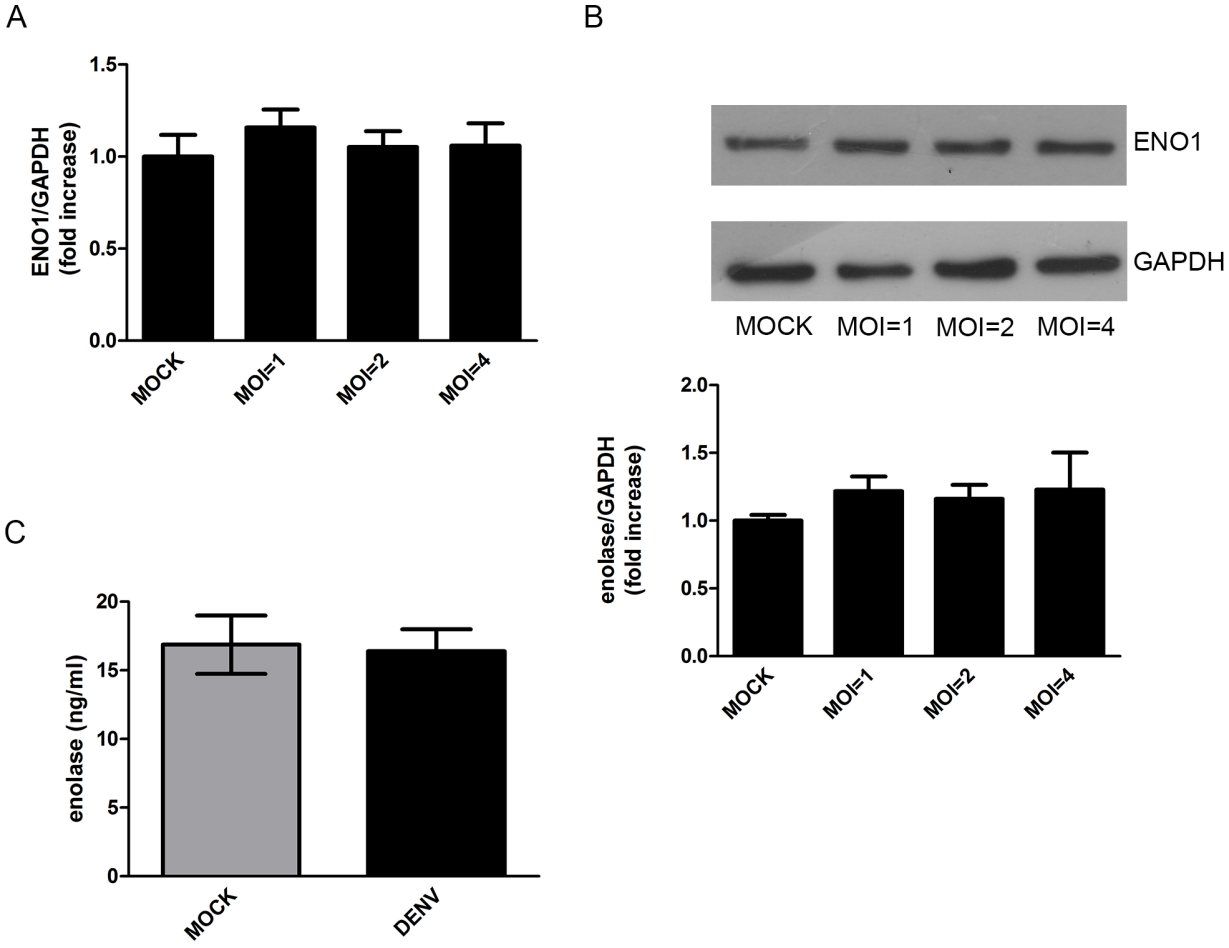
DENV infection does not modulate α-enolase gene expression and intracellular content in HepG2 cells. HepG2 cells were either mock-infected or infected with DENV (MOI = 1, 2 and 4) for 24 h. (A) ENO1 expression was measured by quantitative RT-PCR. Graph shows mean ± SEM from 5 independent experiments. (B) Western blot analysis of cell lysates proteins. Upper panel shows a representative blot and bottom panel shows the densitometric analysis of α-enolase bands normalized by the content of GAPDH. Graph shows mean ± SEM from 3 independent experiments. Comparison between mock and DENV-infected cells was analyzed by repeated measures ANOVA. (C) α-enolase content in cell lysates was measured by ELISA. Graph shows mean ± SEM from 3 independent experiments. Differences between samples from mock and DENV-infected cells were assessed by paired t test.

To investigate whether the increase in α-enolase secretion by DENV-infected cells was a consequence of an enhanced expression of ENO1 gene or due to a virus-induced regulation of α-enolase secretion process, we evaluated the effects of infection on α-enolase mRNA levels and protein intracellular content. Quantitative RT-PCR analyses of mock-infected and cells incubated with DENV at MOI of 1, 2 and 4 for 24 h revealed no differences in ENO1 expression between mock and infected cells (Fig. 4A). Consistently, Western-blot analyses of cell lysates demonstrated that intracellular content of α-enolase is not modulated by DENV infection (Fig. 4B). Finally, ELISA analysis of cellular extracts showed that HepG2 cells infected with DENV at MOI = 4 and mock-infected cells presented similar α-enolase protein content (Fig. 4C). These results indicate that the modulation α-enolase secretion during DENV infection is a post-translational event.

**Figure 5:**
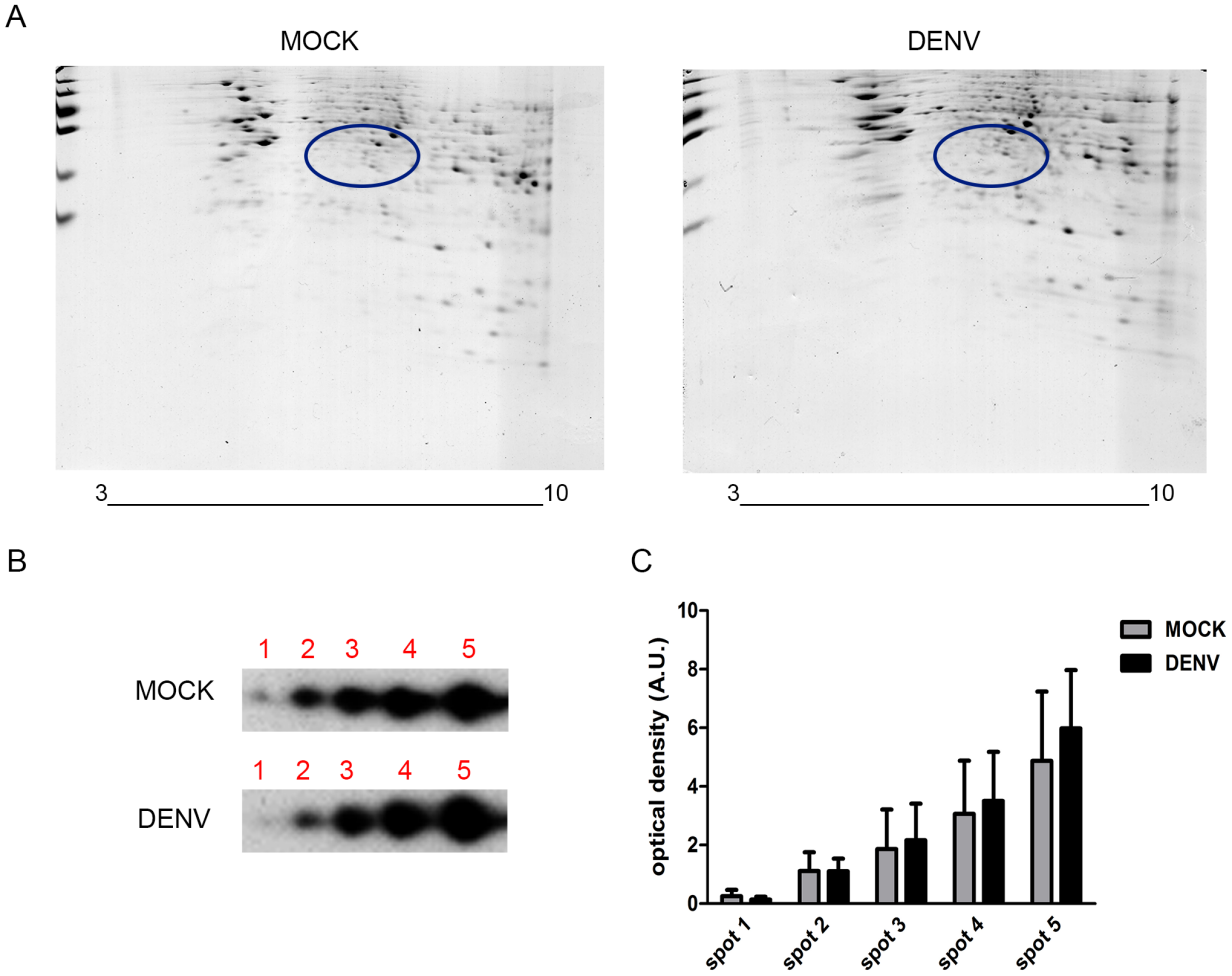
DENV infection does not affect the distribution of intracellular α-enolase isoforms. HepG2 cells were either mock-infected or infected with DENV (MOI = 4) for 24 h. Cell lysates proteins (80 µg) were analyzed by two-dimensional gel electrophoresis (2-DE). (A) Gels stained with colloidal Coomassie blue. The circles indicate the region for α-enolase separation which was selected accordingly to the theoretical molecular mass and isoeletric point of the protein. (B) Immunodetection of α-enolase isoforms on the 2-DE Western blot. α-enolase spots were detected in the region correspondent to the circles in Figure 5A. The figure shows a representative blot of 4 independent experiments. (C) Densitometric analysis of α-enolase spots. Graph shows mean ± SEM from 4 independent experiments. Differences between samples from mock and DENV-infected cells were assessed using repeated measures two-way ANOVA.

To investigate whether DENV infection modulates the distribution of the intracellular isoforms of α-enolase as observed for the secreted isoforms, we performed Western blot analysis (Fig. 5B) from two-dimensional gels of cell lysate samples from mock and DENV-infected cells (Fig. 5A). We observed five spots corresponding to α-enolase with the same pattern as observed for conditioned medium samples (Fig. 3B), i.e., spots with similar molecular weights but distinct isoeletric points. Comparative analysis of the corresponding spots from mock and infected cells revealed no differences in the distribution pattern of the isoforms (Fig. 5C). These results combined with those shown in Fig. 3 suggest that basic isoforms (spots 4 and 5) are addressed to secretion.

**Figure 6:**
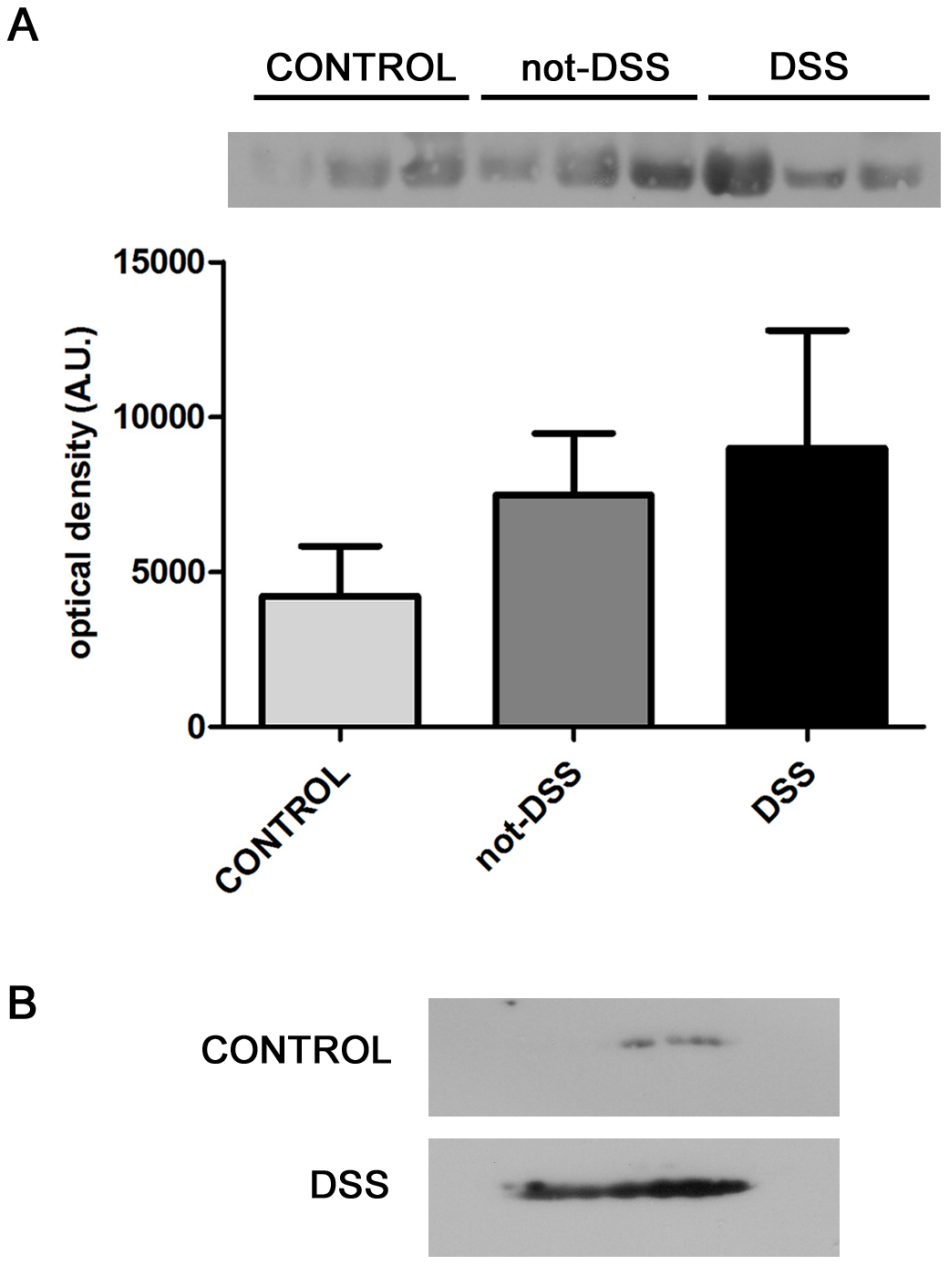
α-enolase levels are altered in plasma samples from dengue patients. Albumin-depleted plasma samples were used to analyze α-enolase content and distribution of protein isoforms. (A) Western blot analysis of albumin-depleted plasma samples from healthy donors (control), non-severe dengue (not-DSS) and dengue shock syndrome (DSS) patients. Upper panel shows blot image and bottom panel shows the densitometric analysis of α-enolase bands. Graph shows mean ± SEM. (B) Albumin-depleted plasma samples (80 µg) were analyzed by two-dimensional gel electrophoresis (2-DE) followed by immunodetection of α-enolase isoforms on the 2-DE Western blot.

To provide insight into the physiological relevance of α-enolase secretion in dengue diseases, we conducted preliminary experiments in which we analyzed α-enolase in albumin-depleted plasma samples from three healthy donors (control), three patients with non-severe dengue (not-DSS) and three patients with dengue shock syndrome (DSS). The results indicated an increase in α-enolase content in plasma of dengue patients (Fig. 6A). To compare the distribution of α-enolase isoforms between plasmas from a healthy donor and a DSS patient, we performed two-dimensional electrophoresis followed by Western blot (Fig. 6B). The result revealed an alteration in the distribution profile of the α-enolase spots in these samples. Although a comprehensive study with larger number of subjects is required to validate these results, our data suggest an interesting potential relevance for α-enolase role in dengue diseases.

## Discussion

Comprehension of the effects of DENV infection on its target cells might provide important insights into dengue pathogenesis. Among the main cells infected by DENV in humans, hepatocytes play a central role [29]. Several data obtained using liver specimens from fatal cases of dengue, animal models, and in vitro cell cultures show that liver is a major site for DENV replication [30]–[34]. Also, liver enlargement and increase in the levels of plasma transaminases are among the criteria to diagnose severe dengue [4]. Additionally, liver produces most of the plasmatic proteins and secretes several inflammatory mediators, which would not only affect organ function but also cause systemic effects, contributing to the increase in vascular permeability as well as to the coagulation disturbs that characterize severe cases of dengue. In this study, we report that DENV infection modulates α-enolase secretion by hepatic cells in a dose-dependent manner. For the first time, we show a positive correlation between DENV infection and secretion of α-enolase, indicating that a high viral load will lead to a burst of α-enolase secretion in hepatic cells. Alterations in α-enolase expression are frequently observed in many pathological conditions. Indeed, in a meta-analysis study that evaluated 169 two-dimensional electrophoresis based articles, α-enolase was identified as the second most often differentially expressed protein in humans, regardless of the tissue analyzed and experimental conditions used [35]. Furthermore, it was the most frequently identified protein with altered expression in mouse and rats. The authors proposed that α-enolase acts as a common stress response protein [35]. Up-regulation of α-enolase expression has been extensively reported for several types of cancer, including hepatocellular carcinoma, thyroid oncocytoma, lung and breast cancers among many others, and ENO1 gene expression is increased in 18 of 24 cancers classes analyzed [36]. In some cases, overexpression of α-enolase positively correlated with tumor progression and clinical outcome [37]–[38]. Additionally, altered levels of α-enolase were also found in Alzheimer's disease, rheumatoid arthritis and systemic sclerosis [20], [39]. Alterations in α-enolase expression were also reported for flaviviruses' infections. α-enolase is one of the up-regulated proteins in the midguts of *Aedes aegypti* mosquitoes and in the *Aedes albopictus* C6/36 cell line infected with different serotypes of DENV [40]–[41]. Interestingly, α-enolase interacts with different viral proteins during the infection, including the capsid protein from DENV, envelope protein from WNV, NS3 protein from Kunjin virus (a variant of WNV) and NS5 protein from Tick-borne encephalitis virus and from Alkhurma virus [42]–[43], although the biological relevance of these interactions remains unclear.

Previously considered a cytoplasmic protein, α-enolase has also been identified in nucleus, plasma membrane, endosome, Golgi complex, peroxisome and in extracellular exosome vesicles [24], [44]–[46]. It is noteworthy that enolase lacks signal sequences for any specific subcellular localization, and the molecular mechanisms involved in its membrane association as well as in its secretion are unknown. The subcellular localization of α-enolase is also modulated in different pathological states. For instance, LPS induces the translocation of α-enolase from cytoplasm to plasma membrane in monocytes [47]. Also, an increase in α-enolase secretion has been observed in response to HIV-1 infection in macrophages [48] and to DENV infection in hepatic cells, as reported here and in our previous study [18]. Alterations in the secretion of α-enolase by liver cells could be involved not only in local effects, as cellular infiltration and hemorrhage reported in liver from patients who died from dengue, but also influence in systemic effects of dengue pathology such as plasma leakage and coagulopathies. Indeed, our preliminary results obtained with plasma samples from DENV-infected patients suggest an association between plasma levels of α-enolase and disease severity. These results point to a potential physiological role of α-enolase in dengue diseases.

Another hypothesis for the role of α-enolase in dengue pathogenesis is that the increased amounts of extracellular, and possibly the membrane-associated, α-enolase would elicit humoral and cellular responses due to the production of autoantibodies and activation of T cells [49]. Detection of anti-enolase antibodies has been reported for several inflammatory and autoimmune diseases, such as pancreatic cancer, lupus erythematosus, rheumatoid arthritis and inflammatory bowel disease [50]–[53]. In addition, the presence of autoantibodies specifically to phosphorylated and citrullinated isoforms of α-enolase has been detected in serum from patients with pancreatic cancer and rheumatoid arthritis, respectively [54]–[55]. Anti-enolase antibodies induced apoptosis in endothelial cells and production of inflammatory mediators by monocytes and macrophages, including TNF-α, IL-1β and IFN-γ [56]–[57], which are also increased in the plasma of dengue patients [58].

The most interesting finding reported here was the modulation of the distribution of α-enolase isoforms secreted by HepG2 cells. Five spots with similar molecular weights but distinct isoeleteric points were identified as α-enolase in the conditioned medium of HepG2 cells, indicating that at least five isoforms of this protein are secreted by these cells. The differences in these isoforms can be explained by the occurrence of post-translational modifications (PTMs) that alter the charge of side chains of the amino acid residues, such as the attachment of charged molecules to neutral residues (phosphorylation) or the addition of functional groups to charged residues (acetylation or methylation). A pattern of α-enolase isoforms very similar to that observed in our study was described in a proteomic analysis of pancreatic ductal cells [28]. In that study, mass spectrometry analysis of the six spots identified as α-enolase showed several PTMs, namely the phosphorylation of one Ser residue, the acetylation of 26 Lys residues and the methylation of 21 Glu and 13 Asp residues [28]. Furthermore, proteolysis of each of the spots resulted in both the post-translationally modified peptides and their unmodified counterparts, suggesting that a myriad isoforms of α-enolase may arise from these combinations of PTMs. These observations suggest that a complex pattern of PTMs is also present in α-enolase secreted by hepatic cells. Additionally, the occurrence of other PTMs in enolase from HepG2 cells could not be ruled out since citrullination, Tyr and Thr phosphorylation, carbonylation, Tyr nitration, Cys glutathionylation and Lys malonylation have been also reported for α-enolase in different tissue specimens and experimental models [59]–[66].

In this work, we showed for the first time that DENV infection not only increases the amount of α-enolase secreted by hepatic cells, but also shifts the distribution of isoforms towards the basic forms, indicating that infection modulates α-enolase PTMs. Modulation of α-enolase PTMs has been observed in other pathologies. In tumor cells, α-enolase shows more PTMs than those occurring in normal tissues, and some particular modifications, such as acetylation, methylation and phosphorylation in specific residues, appear to be associated with cancer development [26], [28]. In addition, increase in citrullinated forms of α-enolase has been reported in brain specimens of patients who died with Creutzfeldt-Jacob or Alzheimer's diseases [59]. Moreover, carbonylation, Tyr nitration and Cys glutathionylation were also α-enolase PTMs observed in Alzheimer's disease [39], [62], [64]–[65].

The role of the alteration in α-enolase isoform pattern during DENV infection and disease progression is a very interesting issue that requires further investigation. PTMs are known to modulate protein stability and activation, interfere with the catalytic activity of enzymes, determine cellular localization of proteins or address them for degradation, as well as regulate protein interactions with different types of ligands [67]. Thus, PTMs determine the protein biological outcomes and orchestrate their role in different processes. However, in the case of α-enolase, very few studies assessed the effects of PTMs on its functions. Studies using rat cardiac muscles revealed that α-enolase enzymatic activity increases in alkaline phosphatase-treated samples, suggesting that phosphorylation has an inhibitory effect on its catalytic activity [68]. On the other hand, increase in α-enolase carbonylation was accompanied by a decrease in its enzymatic activity in mouse models of Huntington's disease [63]. Jang and co-workers showed that citrunilation of α-enolase increases its binding affinity to plasminogen, suppresses enolase catalytic activity and renders enolase more susceptible to degradation by calpain-1 [59]. Since we demonstrated that differences in isoform distribution pattern is observed for the secreted forms but is not detected in cellular extracts, our data support that α-enolase PTMs induced by DENV infection would be involved in the regulation of its subcellular localization in the hepatocytes, specifically addressing the protein to secretion.

The plasminogen binding property of α-enolase makes this protein a putative modulator of intravascular and pericellular fibrinolytic systems. Indeed, the importance of α-enolase as a component of fibrinolytic system has been confirmed by the inhibition of plasminogen activation when cells were incubated with monoclonal anti-α-enolase antibodies [69]. Plasmin is a Ser-protease with a broad spectrum of substrates, which include fibrin, the best characterized substrate, and extracellular matrix components laminin and fibronectin. Plasmin also activates other proteolytic systems including matrix metalloproteinases and collagenase [20]. Therefore, plasminogen activation often leads to extracellular matrix degradation. Extracellular matrix degradation induced by enolase-associated plasmin activity was shown to mediate macrophage infiltration, muscle regeneration, tumor metastasis and pathogen invasion [47], [69]–[73]. In this scenario, it is plausible to speculate that the specific subset of α-enolase isoforms secreted by DENV-infected hepatic cells would be involved in the promotion of fibrinolysis and in the alterations of vascular permeability through extracellular matrix degradation, both processes clearly associated to the pathogenesis of severe dengue.
